# E-cigarette specialty retailers: Data to assess the association between retail environment and student e-cigarette use

**DOI:** 10.1016/j.dib.2016.12.022

**Published:** 2017-01-07

**Authors:** Georgiana Bostean, Catherine M. Crespi, Patsornkarn Vorapharuek, William J. McCarthy

**Affiliations:** a Department of Sociology, Environmental Science & Policy Program, Chapman University, One University Drive, Orange, CA 92866, USA; b Department of Biostatistics, UCLA Fielding School of Public Health, 650 Charles E. Young Dr. South, 51-254 CHS, Los Angeles, CA 90095-1772, USA; c Environmental Management, University of San Francisco, Harney Science Center, 2130 Fulton Street, San Francisco, CA 94117-1080, USA; d UCLA Fielding School of Public Health, Center for Cancer Prevention & Control Research, A2-125 CHS, 650 Charles Young Drive, Los Angeles 90095-6900, USA

**Keywords:** Electronic cigarettes, Retailers, Proximity, Environment, Policy

## Abstract

The retail environment is a major social determinant of health, yet little is known about the e-cigarette specialty retailer environment. The e-cigarette specialty retail environment may be associated with e-cigarette use by middle and high school students, an issue that was addressed in a recent article entitled, “E-cigarette use among students and e-cigarette specialty retailer presence near schools,” by Bostean and colleagues (G. Bostean, C.M. Crespi, P. Vorapharuek, W.J. McCarthy, 2016 [1]). We present data relating to e-cigarette specialty retailers in Orange County, California. We describe the data collection method (including the search methodology to identify e-cigarette specialty retailers), present descriptive retailer data including school proximity, and provide data from multi-level regressions predicting individual-level student use of e-cigarettes based on presence of an e-cigarette specialty retailer in proximity to schools.

**Specifications Table**

**Table:** 

Subject area	*Sociology, Public Health, Policy*
More specific subject area	*Policy, ecological models*
Type of data	*Table, figure*
How data was acquired	*Primary data collected through online search methodology*
Data format	*Analyzed*
Experimental factors	*N/A*
Experimental features	*N/A*
Data source location	*Orange County, California*
Data accessibility	*Data for vape store and school locations accessible.*

**Value of the data**

• The descriptive data presented here for Orange County, CA can be compared to other counties for further insight into regional differences in e-cigarette specialty retailer environment.
• Regressions provide data about the distances to schools that are significantly associated student use of e-cigarettes.
• These data provide useful methodological information that will facilitate future studies of e-cigarette specialty retailer environment.

## 1 Data

E-cigarettes are sold by various retailers, such as liquor stores, convenience stores, and gas stations, and even on the internet [2]. E-cigarette specialty retailers (i.e., “vape stores”) have emerged in many places, yet are understudied. Few studies have attempted to identify in order to assess and monitor the e-cigarette specialty environment.

A recent study examined the association between the presence of e-cigarette specialty retailers near schools and individual middle and high school student use of e-cigarettes [3]. The study focused on Orange County (OC), CA, an ethnically and socioeconomically diverse California county where ever use of e-cigarettes is higher than that of conventional cigarettes [4]. Prior to 2016, OC localities had few tobacco control regulations or e-cigarette regulations beyond the state law prohibiting the sale of e-cigarettes to minors [5].

**Fig. 1:**
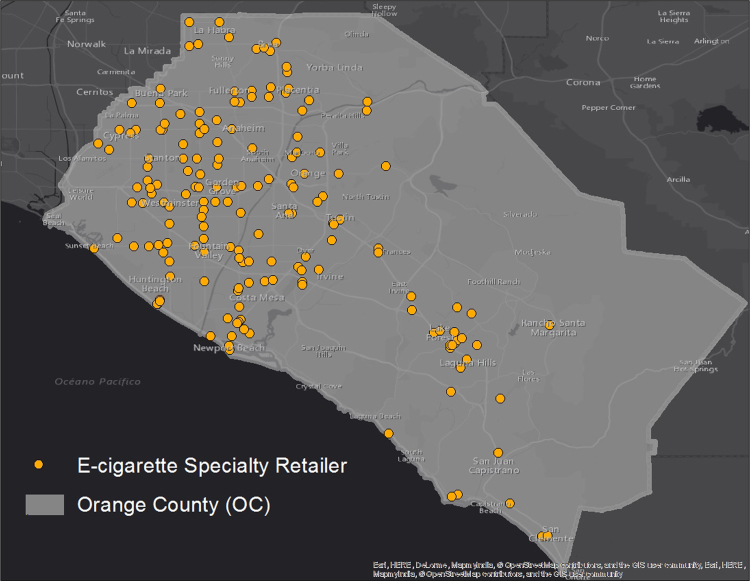
Locations of e-cigarette specialty retailers (vape stores) in Orange County, California.

We present a map of the distribution of retailers (Fig. 1), and descriptive data on e-cigarette specialty retailer environment in OC and how it relates to school locations (Table 1). We also present results from multi-level regressions that examine the effect of e-cigarette specialty retailer presence on individual-level e-cigarette use among middle and high school students; specifically, we present data from three buffer distances (¼ mile, ½ mile, one mile) of schools, which can help guide future research on retailer proximity (Table 2).

## 2 Study design and methods

### 2.1 Methods for assessing e-cigarette specialty retailer environment

#### 2.1.1 Data

We compiled addresses for e-cigarette retailers in OC using a systematic internet search conducted during September 2014-March 2015 (as part of the Orange County E-cigarette Retailer Study). This online search methodology has been reported to be a valid and useful method by which to identify e-cigarette retailers in areas where there is no systematic licensure [6]. Using search engines including Google, Yelp, and Yellowpages, three trained researchers conducted searches using the terms: “Orange County” and “electronic cigarettes,” “e-cigarettes,” or “vape.” This search yielded 173 distinct locations, which were successfully geocoded using ArcMap 10.3 software. The final geocoded locations are visualized in Fig. 1.

To understand the e-cigarette specialty retailer environment, particularly in relation to school locations, we combined the specialty retailer data with location data for schools. Addresses for all public schools were obtained from the California Department of Education [7], [8]. We limited our sample to public schools in OC that are listed as offering grade 7 or higher (*n*=213), regardless of whether the school is an alternative, continuation, or other non-traditional school. We geocoded these 213 school addresses in ArcMap 13.

#### 2.1.2 Measures and analyses

We then conducted buffer analyses to examine the count of schools in proximity to retailers, as well as the count of retailers in proximity to middle and high schools (the latter measure was used in the regressions presented below). Table 1 presents descriptive data about the retailers, including the count and percentage of e-cigarette retailers that have at least one middle or high school within a ¼ mile, ½ mile, and one mile buffer.

Of the public schools analyzed, 6.6% had at least one vape store within a quarter-mile distance of the school, 27.7% had one within one-half mile, and 63.8% had a vape store within one mile of the school. Turning to the vape stores, 8.7% of vape stores are within one-quarter mile of a public school that offers grade 7 (or higher), while 27.2% are within one-half mile and nearly 80% are within one mile of a school. To summarize, the majority of OC vape stores are located within one mile of a public school that offers grade 7 (or higher).

### 2.2 Methods for assessing association between retailer presence in proximity to schools and student e-cigarette use

#### 2.2.1 Data

For the purposes of analyzing the association of student e-cigarette use with specialty retailer proximity to schools, we limited our analysis to e-cigarette specialty retailers confirmed to be open for business during the survey period (September 2013–June 2014); this information was ascertained by calling the retailer or through internet searches. For example, some Yelp reviews document that the location is permanently closed. If that was not available, we searched other websites with business license information through Google search engine. In all, we documented 148 e-cigarette specialty retailers in OC that were open during the survey period. A stratified random sample of 36 retailers were visited for ground truthing.

Student-level data came from the 2013–2014 California Healthy Kids Survey, the largest statewide school survey of risky behaviors in the US. The survey is conducted by WestEd, under contract to the California Department of Education (CDE), and is only available to researchers with a data access and confidentiality agreement. WestEd is a nonprofit educational research and development agency [9]. The survey was designed to be administered at least once every two years to middle school and high school students, grades 7, 9 and 11, attending California public schools and to provide each district with a representative tobacco use profile of its students. School staff administered the survey using detailed instructions provided by WestEd. Participation was voluntary, anonymous, and confidential, and parental consent was obtained. For the CHKS study, all respondents attended OC public middle or high schools (including charter schools, excluding continuation and other non-traditional schools) in grades 7, 9, and 11. We excluded 2621 respondents with missing data on key variables; this represented 3.7% of the sample, which is below the recommended 5% threshold for imputation [10]. The final analytic sample consisted of 67,701 respondents in 130 schools.

#### 2.2.2 Measures and analyses

We combined the school-level and individual-level student data for statistical analysis in Stata 13 software. The outcome of interest was e-cigarette use. *Lifetime e-cigarette use* was defined as self-reported use of e-cigarettes one or more times in one׳s life.

We included school-level and individual-level covariates in our analyses. At the school-level, we included *Free/reduced price lunch program eligibility (FRLP)* for the 2013–2014 academic year. This measure represents the percentage of K-12 students in a school who are eligible for the free or reduced price lunch program and is commonly used as a proxy for school-level SES [11]. At the individual level we included: *gender*; *race-ethnicity* (non-Hispanic White = reference; Hispanic; Black; Asian; Other); *parent׳s education* (less than college = reference; college graduate; don’t know/missing). To avoid excluding the 20% of students with missing information on parent׳s education, “missing/don’t know” was included as a category. We also controlled for several self-reported behavioral risk factors: *Tobacco ever use* assessed whether the student had ever smoked conventional cigarettes or used smokeless tobacco (yes/no); *Alcohol ever use* indicated whether the respondent had ever had a full alcoholic drink (yes/no); *Marijuana ever use* indicated whether the respondent had ever tried marijuana (yes/no).

Multi-level logistic regressions, stratified by school level (middle or high school), predicted presence of retailer within a certain proximity to the school. Three models are presented, each including a different proximity variable (¼ mile, ½ mile, and one mile), controlling for other school-level and individual-level factors. The regression equation is as follows:

$$\log\left(\frac{\pi_{\mathrm{ij}}}{1-\pi_{\mathrm{ij}}}\right)=\beta_{0}+\beta_{1}{\mathrm{Proximity}}_{j}+\sum_{k}^{\;}{Ɣ}_{k}X_{\mathrm{ijk}}+\sum_{l}^{\;}\theta_{l}X_{\mathrm{jl}}+u_{j}$$

where *i* indexes individual, *j* indexes school, $\pi_{\mathrm{ij}}$ is the probability of lifetime (or current) e-cigarette use for individual *i* at school *j*, ${\mathrm{Proximity}}_{j}$ equals 1 for presence of any retailers within the buffer around school *j* and 0 for absence, $X_{\mathrm{ijk}}$ are individual-level covariates, $X_{\mathrm{jl}}$ are school-level covariates, and $u_{j}$ is a normally distributed random intercept with mean zero.

Odds ratios from these regression models are presented in Table 2, and provide information on the retailer distance from schools most relevant for e-cigarette use.

Results show that in this study, the ¼ mile distance is the only distance with a statistically significant effect on individual lifetime e-cigarette use, and only among middle school students. In addition, lower school-level SES (percent of students eligible for free/reduced lunch program) is associated with greater odds of e-cigarette use among middle school students, and individual-level sociodemographic characteristics, including sex, race-ethnicity, parental education, and substance use are also associated with e-cigarette use.

## Figures and Tables

**Table 1:** Descriptives: E-cigarette specialty retailer (Vape Store) environment.

	**Count (*N*)**	**Frequency (%)**
Vape stores within a distance of		
1/4 mile of a school	15	8.7
1/2 mile of a school	47	27.2
1 mile of a school	137	79.2
Total count of vape stores	173	100

Schools within a distance of		
1/4 mile of vape store	14	6.6
1/2 mile of vape store	59	27.7
1 mile of vape store	136	63.8
Total count of middle and high schools	213	100

*Note:* Data are for Orange County, CA. Table shows count and frequency of vape stores within specified distance of at least one school, and schools within a specified distance of at least one vape store.

**Table 2:** Multilevel logistic regression odds ratios predicting lifetime e-cigarette use among middle and high school students in Orange County, CA.

	**Middle School** (*n*=23,091)		**High School** (*n*=44,610)			
	OR (95% CI)		OR (95% CI)			
***School-level predictors***						
Retailer presence (ref=none within 1/4 mile)	1.70⁎	–	–	1.02	–	–
	(1.02–2.83)			(0.81–1.29)		
						
Retailer presence (ref=none within 1/2 mile)	–	1.20	–	–	1.02	–
		(0.91–1.60)			(0.87–1.19)	
						
Retailer presence (ref=none within one mile)	–	–	1.00	–	–	1.10
			(0.78–1.28)			(0.93–1.29)
						
% eligible for free/reduced lunch program	1.01⁎⁎⁎	1.01⁎⁎⁎	1.01⁎⁎⁎	1.00	1.00	1.00
	(1.01–1.01)	(1.01–1.01)	(1.01–1.02)	(1.00–1.00)	(1.00–1.00)	(1.00–1.00)
						
***Individual-level predictors***						
Female (ref=Male)	0.84⁎⁎⁎	0.84⁎⁎⁎	0.84⁎⁎⁎	0.80⁎⁎⁎	0.80⁎⁎⁎	0.80⁎⁎⁎
	(0.76–0.93)	(0.76–0.93)	(0.76–0.93)	(0.75–0.85)	(0.75–0.85)	(0.75–0.85)
						
Race-ethnicity (ref=non-Hispanic White)						
Hispanic	1.54⁎⁎⁎	1.54⁎⁎⁎	1.54⁎⁎⁎	1.07	1.07	1.07
	(1.28–1.85)	(1.28–1.86)	(1.28–1.86)	(0.98–1.16)	(0.98–1.16)	(0.98–1.16)
						
Black	1.06	1.06	1.07	0.84	0.84	0.84
	(0.65–1.74)	(0.65–1.74)	(0.65–1.74)	(0.66–1.08)	(0.66–1.08)	(0.65–1.08)
						
Asian	0.57⁎⁎⁎	0.57⁎⁎⁎	0.57⁎⁎⁎	0.69⁎⁎⁎	0.69⁎⁎⁎	0.69⁎⁎⁎
	(0.44–0.74)	(0.44–0.74)	(0.45–0.74)	(0.62–0.77)	(0.62–0.77)	(0.62–0.77)
						
Other	1.31⁎	1.31⁎	1.31⁎⁎	1.11+	1.11+	1.10+
	(1.07–1.61)	(1.07–1.61)	(1.07–1.61)	(1.00–1.22)	(1.00–1.22)	(1.00–1.22)
						
Parent education (ref=College grad)						
< College graduate	1.44⁎⁎⁎	1.44⁎⁎⁎	1.44⁎⁎⁎	1.26⁎⁎⁎	1.26⁎⁎⁎	1.26⁎⁎⁎
	(1.24–1.66)	(1.24–1.66)	(1.24–1.66)	(1.17–1.35)	(1.17–1.35)	(1.17–1.35)
						
Missing/Don׳t know	1.23⁎⁎	1.23⁎⁎	1.23⁎⁎	1.20⁎⁎⁎	1.20⁎⁎⁎	1.20⁎⁎⁎
	(1.06–1.42)	(1.06–1.42)	(1.06–1.42)	(1.09–1.31)	(1.09–1.31)	(1.09–1.31)
						
Tobacco ever use (ref=never used)	6.84⁎⁎⁎	6.84⁎⁎⁎	6.86⁎⁎⁎	5.02⁎⁎⁎	5.02⁎⁎⁎	5.02⁎⁎⁎
	(5.61–8.34)	(5.62–8.34)	(5.63–8.36)	(4.63–5.43)	(4.63–5.43)	(4.63–5.44)
						
Alcohol ever use (ref= never had alcohol drink)	5.82⁎⁎⁎	5.82⁎⁎⁎	5.82⁎⁎⁎	5.19⁎⁎⁎	5.19⁎⁎⁎	5.19⁎⁎⁎
	(5.17–6.56)	(5.17–6.56)	(5.17–6.55)	(4.87–5.53)	(4.87–5.53)	(4.87–5.53)
						
Marijuana ever use (ref= never used)	8.15⁎⁎⁎	8.14⁎⁎⁎	8.15⁎⁎⁎	4.83⁎⁎⁎	4.83⁎⁎⁎	4.83⁎⁎⁎
	(6.90–9.62)	(6.90–9.60)	(6.90–9.61)	(4.52–5.15)	(4.52–5.15)	(4.52–5.15)
						
*n*	23,091	23,091	23,091	44,610	44,610	44,610
Number of groups	123	123	123	83	83	83

*Notes:*

Odds ratios derived from multilevel logistic regressions (full model shown). Middle school includes grade 7, high school includes grades 9 and 11. % of students within school eligible for free/reduced lunch program (FRLP).

⁎ *p*<.05.

⁎⁎ *p*<.01.

⁎⁎⁎ *p*<.001.
